# Long-term iron supplementation in four patients with X-linked erythropoietic protoporphyria: associations with serum proteins and erythrocyte protoporphyrin levels—a single-centre retrospective study

**DOI:** 10.3389/fmolb.2025.1509803

**Published:** 2025-02-13

**Authors:** Anna-Elisabeth Minder, Francesca Granata, Franziska van Breemen, Xiaoye Schneider-Yin, Elisabeth I. Minder, Lanja Saleh, Jasmin Barman-Aksözen

**Affiliations:** ^1^ Division of Endocrinology, Diabetology, and Porphyria, Stadtspital Zürich Triemli, Zurich, Switzerland; ^2^ Swiss Reference Centre for Porphyrias, Stadtspital Zürich, Triemli, Zurich, Switzerland; ^3^ Fondazione IRCCS Ca’ Granda Ospedale Maggiore Policlinico, S.C Medicina ad Indirizzo Metabolico, Milano, Italy; ^4^ Institute of Laboratory Medicine, Stadtspital Zürich, Triemli, Zurich, Switzerland; ^5^ Division of Metabolism and Children’s Research Center, University Children’s Hospital, Zurich, Switzerland; ^6^ University Research Priority Program “ITINERARE – Innovative Therapies in Rare Diseases”, University of Zurich, Zurich, Switzerland

**Keywords:** erythropoietic protoporphyria, X-linked erythropoietic protoporphyria, iron, safety, effectiveness, treatment, ALAS2

## Abstract

**Introduction:**

X-linked erythropoietic protoporphyria (XLEPP) is an ultra-rare inborn error of the heme biosynthesis characterised by the accumulation of large amounts of protoporphyrin IX (PPIX) and zinc-protoporphyrin in the erythrocytes. PPIX absorbs the energy of the visible light range and upon exposure causes painful phototoxic reactions and tissue damage. In addition, PPIX is excreted via the liver and bile, and can induce liver failure that requires life-saving liver transplantation. Case reports and data from a limited number of patients enrolled in a prospective study indicate that supplementation with iron, a co-substrate of the heme biosynthesis, can decrease blood PPIX concentrations and improve liver damage and photosensitivity in patients with XLEPP. However, long-term data on safety and effectiveness of iron supplementation in XLEPP to support this treatment strategy is limited.

**Methode:**

Here, we report the experience and long-term effects over up to 8 years of iron supplementation of the four patients with XLEPP in the Swiss cohort.

**Results:**

Our study shows that iron supplementation was safe and effective in lowering blood PPIX concentrations in our patients in the long term.

**Discussion:**

However, monitoring for adequate dosing and long-term effects is advisable and a standardisation of treatment protocols and international best practice guidelines are needed.

## 1 Introduction

**FIGURE 1:**
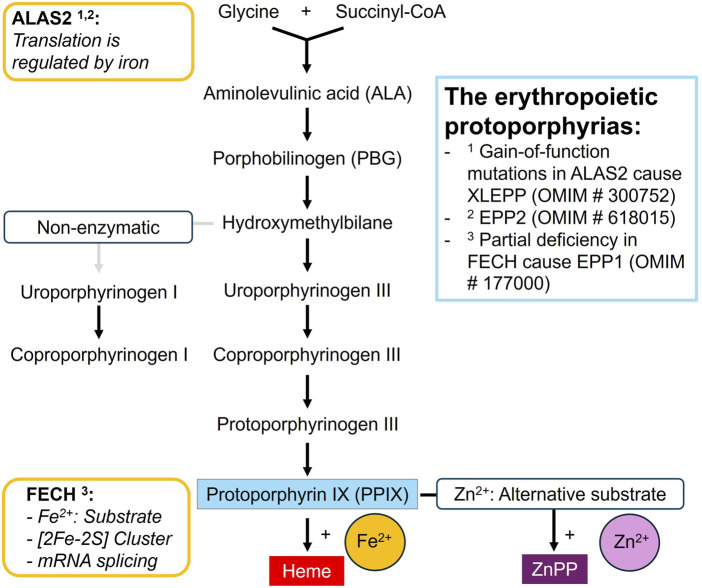
The erythroid heme biosynthesis pathway.

Iron is essential for life, yet it is a highly reactive and potentially toxic substance. Therefore, its absorption, utilisation and distribution in the body is strictly regulated (Abbaspour et al., 2014; Yuen and Becker, 2023; Belot et al., 2024). One of the major demands for iron in the body is created by the erythropoietic heme biosynthesis to produce hemoglobin (Belot et al., 2024). Heme synthesis occurs in eight consecutive steps: The substrates glycine and succinyl-CoA are initially converted to delta-aminolevulinic acid (ALA) and further processed into the monopyrrol porphobilinogen, which serves as the building block for the porphyrin ring (Figure 1). In the final step, ferrochelatase (FECH) incorporates ferrous iron into the last precursor, protoporphyrin IX (PPIX), to form heme. In case of iron deficiency, FECH uses its alternative substrate, zinc (Zn), to form zinc-protoporphyrin (ZnPP), which, however, cannot replace heme.

Iron not only serves as a substrate, but also forms an essential part of the indispensable iron-sulfur clusters in the eukaryotic FECH and aminolevulinic acid dehydratase (ALAD) enzymes in the heme biosynthetic pathway (Dailey et al., 2000; Belot et al., 2024). In addition, *in-vitro* studies suggest that iron deprivation can lead to decreased FECH enzyme stability and an increase in aberrant splicing of FECH mRNA (Taketani et al., 2000; Crooks et al., 2010; Barman-Aksözen et al., 2013). Most importantly, iron regulates the synthesis rate of porphyrins: Delta-aminolevulinate synthase 2 (ALAS2) acts as the rate-limiting enzyme in the erythroid heme biosynthesis (Bhasker et al., 1993; Melefors et al., 1993; Sadlon et al., 1999; Peoc’h et al., 2019). The 5′-untranslated region of ALAS2 mRNA contains a conserved stem look structure known as the iron-responsive element (IRE), which serves as the binding site for the IRE-binding proteins (IRPs) 1 and 2. A low intracellular iron concentration promotes the binding of IRP to the IRE which prevents translation of the mRNA into ALAS2 enzyme and therefore the synthesis of ALA. The IRP-IRE mechanism establishes a direct link between iron availability and the capacity of the erythropoietic heme biosynthetic pathway, ensuring efficient iron utilisation while preventing the accumulation of toxic heme precursors during iron deficiency. Apparently, this fine-tuned balance is disturbed by pathogenic variants in the genes for *FECH* and *ALAS2* which both lead to the accumulation of PPIX and disturbances in the iron metabolism (Poli et al., 2021; Poli et al., 2024).

As first described by Magnus et al. (1961) and further elucidated by Went and Klasen (1984) and Gouya et al. (2002), a partial deficiency in FECH causes EPP1 (OMIM # 177000), which is characterised by the accumulation of large amounts of PPIX in the erythrocytes. PPIX absorbs the energy of the visible light range and upon exposure causes painful phototoxic reactions and tissue damage (Maitra et al., 2019). In addition, PPIX is excreted via the liver and bile, and in 2%–5% of the patients induces liver failure that requires life-saving liver transplantation (Anstey and Hift, 2007; Wahlin et al., 2011a; Wensink et al., 2022). EPP1 is also associated with disturbances in the iron metabolism: while a pronounced anaemia is only present in a minority of the patients, low serum iron, ferritin and transferrin saturation and hypochromic, microcytic erythrocytes are commonly observed and indicate an iron deficiency (Holme et al., 2007; Delaby et al., 2009; Wahlin et al., 2011b; Balwani et al., 2017; Minder et al., 2021). However, iron supplementation, despite moderately increasing blood hemoglobin concentrations, has been shown to increase erythrocyte PPIX concentrations and phototoxicity in most of the investigated patients with a confirmed EPP1 (Balwani et al., 2022; Heersfordt et al., 2024). The observed increase in PPIX under iron supplementation may be linked to elevated expression of ALAS2 mRNA and protein, as measured in peripheral blood samples of patients with EPP1: under iron supplementation, the translational repression of the ALAS2 mRNA is lifted which in turn leads to an increase in ALA synthesis and enhanced PPIX accumulation (Barman-Aksözen et al., 2015; Granata et al., 2022). Interestingly, the exact cause for the iron deficiency in EPP1 patients remains unclear: On the one hand, hepcidin, a hepatic peptide hormone that regulates iron homeostasis and prevents iron uptake und release from the body iron stores during inflammation is not upregulated and therefore does not prevent nutritional iron absorption in EPP1 (Bossi et al., 2015; Barman-Aksozen et al., 2017; Graziadei et al., 2022). On the other hand, in patients with EPP1, no indication for either excessive iron storage or iron loss has been observed so far.

In 2008, Whatley et al. identified gain of function mutations in *ALAS2* exon 11 in a subgroup of patients who were negative for *FECH*-mutations. This discovery led to the characterised of X-linked EPP (XLEPP, OMIM # 300752), which is clinically indistinguishable from EPP1 but is characterised by the additional accumulation of zinc-protoporphyrin (ZnPP) in the erythrocytes. In XLEPP, the overly active ALAS2 enzyme is no longer regulated by iron availability but leads to the overproduction of ALA in maturing erythrocytes which is converted to PPIX (Ducamp et al., 2013; Fratz et al., 2015). It is assumed that iron becomes the limiting factor of the heme biosynthesis in XLEPP and that FECH (which is not affected in XLEPP) utilises its alternative substrate zinc in addition to iron. Case reports and data from three patients enrolled in a prospective study indicate that iron supplementation may decrease blood PPIX concentrations and improve liver damage and photosensitivity in patients with XLEPP (Table 1). More recently, Yien et al. (2017) in one family discovered that mutations in the *CLPX* gene can cause EPP2 (OMIM # 618015). CLPX regulates the expression of ALAS2 and EPP2 resembles the clinical picture of XLEPP.

**TABLE 1:** Previously reported cases of confirmed XLEPP treated with iron supplementation.

References	n	Patient characteristics	Treatment	PPIX baseline	PPIX under treatment	Clinical effect	Follow-up period
Whatley et al. (2008)	1	n.a.	Oral iron	Ca.170 μmol/L ZnPP: ca. 30 μmol/L	Ca. 50 μmol/L ZnPP: ca. 30 μmol/L	- Increase in Hb and serum ferritin	30 months
Sonderup et al. (2009)	2	Male, age 63	Iron i.v.	n.a.	Initial transient increase, followed by a decrease compared to baseline	- Normalisation of liver function tests within 7 days of therapy. -Reduction in photosensitivity	3 months
		Female, age 24	Iron i.v.	n.a.	Initial transient increase, followed by a decrease compared to baseline	- Normalisation of liver function tests within 7 days of therapy. - Reduction in photosensitivity	3 months
Landefeld et al. (2016)	1	Male, age 9, Caucasian	Treatment because of liver decompensation: 12 unites red blood cells (=1800 mg iron) over 2 months −400 mg haem arginate i.v. (=103 mg iron)	94.6 μmol/L, (normal <0.09)	2 μmol/L	- Reversal of liver failure requiring additional 17 RBC transfusions (=2,550 mg iron) and iron chelation therapy - Oral iron as maintenance therapy for 3 years: Normalised liver function test and improved liver biopsy findings, reduction in phototoxicity	3 years
Balwani et al. (2022)	3	Female, Caucasian	325 ferrous sulfate orally twice daily (=130 mg elemental iron per day), 3 months	n.a.	Decrease compared to baseline	n.a.	3 months (early study termination, no follow-up reported after 3 months)
		Female, Caucasian	325 ferrous sulfate orally twice daily (=130 mg elemental iron per day), 12 months	1,705 μg/dL	560 μg/dL	- Reduction in phototoxicity - No adverse events	12 months
		Female, Caucasian	325 ferrous sulfate orally twice daily (=130 mg elemental iron per day), 12 months	2,440 μg/dL	1,218 μg/dL	- Reduction in phototoxicity	12 months

Studies across different cohorts have shown that 2%–10% of patients affected by PPIX accumulation carry gain of function mutations in ALAS2, and that XLEPP is associated with higher blood PPIX concentrations and an increased risk for liver failure as compared to EPP1 (Whatley et al., 2008; Ducamp et al., 2013; Brancaleoni et al., 2016; Balwani et al., 2017; Ventura et al., 2020). Several porphyria treatment centres currently recommend their patients with XLEPP to supplement iron, with anecdotally good results (Di Pierro et al., 2022; Leaf and Dickey, 2023). However, long-term data on safety and effectiveness of iron supplementation in XLEPP to support this advice is still limited. Here, we report the experience and long-term effects of iron supplementation of four patients with XLEPP in the Swiss cohort. For our study, we retrospectively analysed the available laboratory results from the clinical records with the aim to assess long-term safety and effectiveness of iron supplementation in XLEPP and to identify potential markers for the monitoring of the treatment effects.

## 2 Material and methods

### 2.1 Informed consent

Prior to the analysis, all patients signed written informed consent forms for the participation in the porphyria biobank project, which was approved by the Cantonal Ethics Committee in Zurich (BASEC 2018-00758). In addition, a separate written informed consent was obtained from all patients prior to mutational analysis of FECH and ALAS2 genes. All investigations were conducted in accordance with the ethical principles of the Declaration of Helsinki.

### 2.2 Treatment protocol

After the identification of XLEPP in 2008, all patients with negative FECH mutational analysis and/or elevated erythrocyte ZnPP concentrations in the Swiss cohort were reanalysed. From 2016 on, based on beneficial effects reported in the literature, the four XLEPP patients identified in the Swiss cohort were advised to take iron supplements. In case oral iron supplements were not tolerated or efficient, iron infusions were offered. The frequency and dosing were adjusted to the individual patients need after assessing their PPIX, ZnPP, ferritin and hemoglobin concentrations. We usually recommend additional iron supplementation on an individual basis when ZnPP increases to >30% of total PPIX and/or >50% of metal free PPIX and/or in case the liver function tests are increased and/or hemoglobin decreased or ferritin decreased below 50 ug/L, taking the patients wellbeing into account. After establishing a suitable dosing regime and because of the apparent well tolerability of the treatment and to reduce the burden to having to travel to the national reference centre for each appointment, iron supplementation was organised and performed locally with the patient`s GPs. During their routine visits at our treatment centre, parameters for iron and porphyrin metabolism, liver health and haematological parameters are monitored every 6 months. All four patients with XLEPP are in addition treated with afamelanotide for the prevention of phototoxic reactions.

### 2.3 Analyses

All biochemical analyses were performed at the Institute of Laboratory Medicine (ILM) at the Municipal Hospital Zurich as routine analyses. Quantification of erythrocyte porphyrins and DNA sequencing were performed as published earlier (Minder and Schneider-Yin, 2008). Results for ferritin (which is also an acute phase protein) were excluded from the analysis in the case of an elevated c-reactive protein. The ILM is part of the Swiss Reference Centre for Porphyrias and all routine analyses, including the porphyria and genetic analyses, are accredited by the Swiss Accreditation Service (SAS), according to the ISO norm 15,189 for medical laboratories.

### 2.4 Statistical analysis

For correlations, Kendall`s Tau rank correlation test was performed. A correlation coefficient r of 0.4–0.69 was considered as moderate, 0.7–0.89 as strong, and ≥0.9 as a very strong correlation. Data with independent samples were analysed by Mann-Whitney *U* test (R Core Team, 2021). A two-sided p ≤ 0.05 was considered statistically significant.

## 3 Results

### 3.1 Patients included in the study

**FIGURE 2:**
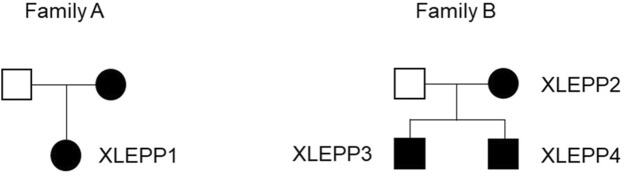
Pedigrees of the two families with XLEPP in the Swiss cohort.

We included n = 4 patients from two families in our study, all having a biochemically and/or genetically confirmed diagnosis of XLEPP (Figure 2; Table 2).

**TABLE 2:** Patient characteristics, baseline laboratory values and time to follow-up since recommendation of iron supplementation.

	Age, and sex	Diagnosis	PPIX (μmol/L) and ZnPP (μmol/L)	Hb (g/dL) and ferritin (μg/L)	Follow-up period since iron supplementation and form of administration
XLEPP 1	49, female	Biochemical and mutational analysis. FECH: negative ALAS2: c.1706–1709delAGTG (p.E569GfsX24)	**22** (<0.2) **13.9** (<1.3)	13 (12–15.4) 24 (10–160)	8 years oral iron
XLEPP 2	61, female	Biochemical and mutational analysis. FECH: negative ALAS2: c.1706–1709delAGTG; (p.E569GfsX24)	**20.5** (<0.2) **4.36** (<1.3)	**11.5** (12–15.4) 41 (10–160)	8 years Iron i.v.
XLEPP 3	34, male	Biochemical	**30.9** (<0.2) **8.6** (<1.3)	14.4 (13.5–17.2) 63 (30–400)	5 years unknown
XLEPP 4	31, male	Biochemical	**48.6** (<0.2) **11.2** (<1.3)	**12.9** (13.5–17.2) **10** (30–400)	8 years Iron i.v.

In bold: pathological parameters.

### 3.2 Safety and effectiveness of iron substitution

The effect of iron substitution was assessed by analysis of the protoporphyrin, hemoglobin and serum ferritin concentrations. In addition, platelet count was analysed (Table 3). Mean and median values of all laboratory parameters can be found in the supplement (Supplementary Tables S1A–D).

**TABLE 3:** Mean change before vs under iron supplementation.

Mean change [%]	PPIX	ZnPP	Hb	Ferritin	Platelets
XLEPP 1	−42.6*	−46.7*	+2.4	+209.8	+11.6
XLEPP 2	**−4.5**	**+1.7**	+6.8*	+113.7	+35.8*
XLEPP 3	**−44.3**	**−5.6**	+1.4	+214.7	+41
XLEPP 4	−64.5*	−36.1*	+15.4*	+978.8	+94.3*

*indicates *p* ≤ 0.05; ** indicates *p* < 0.001; *** indicates p < 0.0001; in bold: parameters which were pathological at baseline and remain pathological; underlined: normalisation of previously pathological parameters.

**FIGURE 3:**
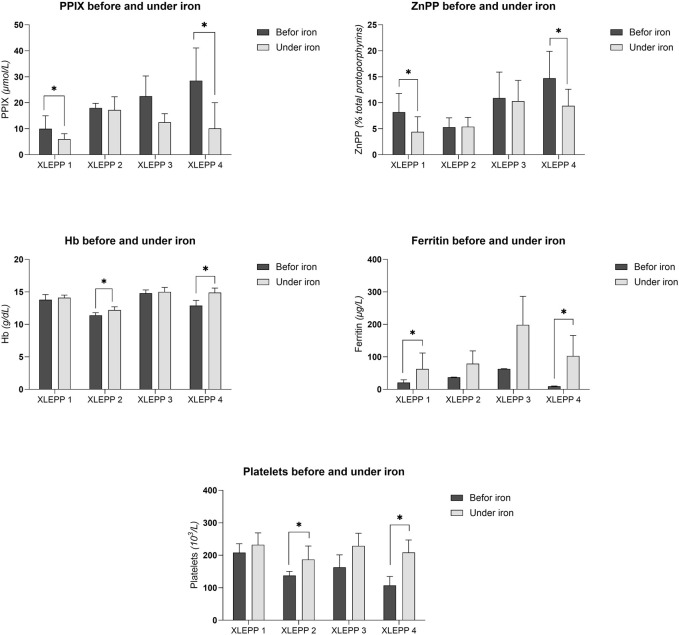
**(A)** Erythrocyte PPIX concentrations. **(B)** Erythrocyte ZnPP concentrations. **(C)** Blood hemoglobin concentrations. **(D)** Serum ferritin concentrations. **(E)** Platelet count.

In all four patients with XLEPP in the Swiss cohort, iron supplementation was accompanied by decreased erythrocyte PPIX concentrations, although only reaching a statistically significant difference in two of the patients (Figure 3A). In addition, a decrease in erythrocyte ZnPP concentrations was observed in 3 of the four patients (Table 3; Figure 3B). Concomitantly, hemoglobin, serum ferritin and platelet count increased, and lead to a normalisation of previously pathological values (Figures 3C–E). Iron stores as assessed by serum ferritin concentrations increased in all patients but remained within the reference ranges with no indication for iron overload. In addition, bilirubin remained within the normal range in all four patients (Supplementary Tables S1A–D). Moreover, in patient 1, pathological liver enzymes normalised under treatment with iron supplementation.

### 3.3 Markers for treatment monitoring

We further assessed which parameters could be used to monitor treatment effects and support dosing decisions (Table 4). In our cohort, platelet counts in three of the four patients inversely and statistically significantly correlated with erythrocyte PPIX concentrations. Moreover, in all four patients, a trend was observed that erythrocyte PPIX concentrations positively correlate with ZnPP and negatively correlate with hemoglobin and ferritin.

**TABLE 4:** Correlations PPIX.

PPIX vs	ZnPP (n)	Hb (n)	Ferritin (n)	Platelets (n)
XLEPP 1	0.3* (32)	−0.01 (31)	−0.6** (15)	−0.5*** (31)
XLEPP 2	0.1 (25)	−0.4* (23)	−0.1 (19)	−0.3* (23)
XLEPP 3	0.3 (11)	−0.3 (10)	−0.4 (9)	−0.4 (9)
XLEPP 4	0.4* (22)	−0.6*** (21)	−0.5* (17)	−0.5** (21)

*indicates *p* ≤ 0.05; ** indicates *p* < 0.001; *** indicates *p* < 0.0001.

## 4 Discussion

In all four of our patients with XLEPP, the recommendation to start iron supplementation resulted in a decrease in mean erythrocyte PPIX concentrations, ranging from 4.5% to 64.5%, as compared to the mean erythrocyte PPIX concentrations before iron supplementation was recommended. Moreover, in all but one patient erythrocyte ZnPP concentrations decreased, too. Interestingly, the mean decrease in erythrocyte PPIX concentrations was more pronounced than the decrease in ZnPP which indicates that limited substrate availability, i.e., an iron deficiency, contributes to the PPIX accumulation in XLEPP. Concomitantly, serum ferritin and blood hemoglobin concentrations increased, but remained within their respective reference ranges without signs for an iron overload throughout the observational period between five to 8 years. Clinically, the patients reported an improvement of their general wellbeing with iron supplementation. In none of the patients a complete freedom from phototoxic reactions was achieved by iron supplementation, which is in accordance with the clinical experience in our treatment centre that erythrocyte PPIX concentration ≥5 μmol/L cause phototoxic reactions.

Importantly, no direct adverse events or other long-term negative effects of iron supplementation were identified in our cohort. However, elevation in liver enzymes in two of the patients warrant further monitoring. Based on the current understanding of the XLEPP disease pathophysiology, decreased blood PPIX concentrations should lower the risk for liver involvement. As currently most experience with iron supplementation is based on short-term treatment of iron deficiency and no active mechanism to excrete iron from the human body is known, the question remains on where the cumulative iron doses end up (Fischer and Naughton, 2004;Fernández-Gaxiola and De-Regil, 2019).

An incidental finding of our study was that two of the patients at baseline showed mild thrombocytopenia (low platelet count), which was resolved under iron treatment. While the interplay between iron and platelet count is not yet completely understood, low platelet count has been described in patients with an absolute iron deficiency (Brissot et al., 2021). However, the exact mechanism and underlying reasons for the low platelet count observed in our patients with XLEPP needs to be further elucidated.

### 4.1 Limitations of the study

One of the main limitations of our study is that the iron supplementation was implemented by the local physicians. Also, patients have access to over-the-counter iron supplements. Therefore, we were not able to determine the exact dose and frequency of iron supplementation the patients were using. Moreover, some patients with XLEPP may have started to use iron to better control their phototoxicity even before the condition had been first described. Especially women in child-bearing age likely have been using iron in order to treat iron deficiency due to menstrual blood loss. Out of the reasons discussed above, our analysis might not reflect the full extent of the effect iron supplementation has on blood PPIX concentrations in XLEPP. However, the correct diagnosis allowed to monitor these patients and adjust the iron doses to their individual needs which might be the reason on why we in all four patients nevertheless observed a decrease in mean PPIX blood concentrations. Prospective study protocols and systematic monitoring of iron intake are required for a definitive assessment.

## 5 Conclusion

Based on our own data and the published and informally shared experiences of the porphyria treatment centres internationally iron supplementation seems to be safe and effective in lowering blood PPIX concentrations in patients with XLEPP also in the long term. However, monitoring for adequate dosing and long-term effects is advisable. Moreover, a standardisation of treatment protocols and long-term experiences is needed. As XLEPP is a subforum of an already ultra-rare disease, data from the single treatment centres should be shared to enable formulation of best practice guidelines.

## Data Availability

The original contributions presented in the study are included in the article/Supplementary Material, further inquiries can be directed to the corresponding author.
