# Supplementary material for: Long-term iron supplementation in four patients with X-linked erythropoietic protoporphyria: associations with serum proteins and erythrocyte protoporphyrin levels—a single-centre retrospective study
Source: Front Mol Biosci. 2025 Feb 13;12:1509803. doi: 10.3389/fmolb.2025.1509803 (PMC11864907; doi:10.3389/fmolb.2025.1509803)
Supplement: Supplementary file 1 [file DataSheet1.PDF]

Supplement

Long-term Iron Supplementation in Four Patients with X-linked Erythropoietic Protoporphyria: Associations with Serum Proteins and Erythrocyte Protoporphyrin Levels—A Single-Centre Retrospective Study

Anna-Elisabeth Minder et al

Laboratory results before and after recommendation for iron supplementation

Table S1a: Laboratory parameters patient XLEPP1

XLEPP1 (f.)	Reference range	Before 2016<br>Mean (SD)<br>Median (range)<br>n	Since 2016<br>Mean (SD)<br>Median (range)<br>n
PPIX	<0.2 µmol/L	**10.4** (5)<br>**9** (4.7-22.4)<br>20	**6** (2.1)<br>**5.1** (3.5-11.4)<br>13
ZnPP	<1.3 µmol/L	**8.2** (3.6)<br>**7.4** (3.7-17.4)<br>20	**4.4** (2.9)<br>**3.8** (1.3-12)<br>12
Relative ZnPP	%	44.5 (10.7)<br>44.3 (30.2-66.1)<br>20	39.3 (10.4)<br>40.7 (22.9-54.4)<br>13
Hb	12.0-15.4 g/dL	13.8 (0.8)<br>13.8 (11.9-15.1)<br>20	14.1 (0.4)<br>14.2 (13.5-14.8)<br>12
Platelets	150-370 10^3 /L	208.3 (27.2)<br>209.5 (186-244)<br>20	232.4 (36.4)<br>241.5 (164-272)<br>12
Eisen	6.6-26 µmol/L	20.8 (11.7)<br>23.4 (5.1-31.2)<br>4	n.a.
Ferritin	10-160 µg/L	20.8 (8.7)<br>22.5 (8-28)<br>4	62.7 (49.3)<br>44 (22-172)<br>11
Transferrin	23-45 µmol/L	36 (2.8)<br>36 (34-38)<br>2	n.a.
TfSat	23-46%	33 (7.1)<br>33 (28-38)<br>2	n.a.
Total bilirubin	<21 µmol/L	13.8 (7.5)<br>12 (8-32)<br>8	6.7 (1.6)<br>6.5 (4-9)<br>10
GOT	<32 IU/L	**60** (148.8)<br>20 (17-577)<br>14	19.8 (3.4)<br>20 (15-25)<br>12
GPT	<33 IU/L	**80.4** (186.1)<br>14 (12-541)<br>8	16.6 (5.4)<br>16 (9-24)<br>10
AP	<117 IU/L	49.1 (34.4)<br>36 (34-127)<br>7	53.3 (12.8)<br>57 (35-69)<br>10
Gamma-GT	5-36 IU/L	14<br>14<br>1	17.4 (4.2)<br>17.5 (10-24)<br>10

Table S1b: Laboratory parameters patient XLEPP2

XLEPP2 (f.)	Reference range	Before 2016<br>Mean (SD)<br>Median (range)<br>n	Since 2016<br>Mean (SD)<br>Median (range)<br>n
PPIX	<0.2 µmol/L	**18** (1.8)<br>**18.2** (15.4-20.5)<br>5	**17.2** (5.1)<br>**16.5** (8.6-30.7)<br>20
ZnPP	<1.3 µmol/L	**5.3** (1.8)<br>**4.5** (4.4-7.9)<br>5	**5.4** (1.8)<br>**5.3** (1.1.-7.9)<br>20
Relative ZnPP	%	22.7 (6.1)<br>22.7 (16.2-31.1)<br>5	24.4 (8.5)<br>24.2 (7.2-48)<br>20
Hb	12.0-15.4 g/dL	**11.4** (0.4)<br>**11.2** (11.1-12.1)<br>5	12.2 (0.5)<br>12.1 (11.7-13.4)<br>18
Platelets	150-370 10^3 /L	**137.8** (12.5)<br>**133** (124-152)<br>5	187.2 (41.3)<br>180.3 (133-326)<br>18
Eisen	6.6-26 µmol/L	10.4<br>1.04<br>1	15.4 (11.3)<br>9.1 (8.7-28.5)<br>3
Ferritin	10-160 µg/L	37 (0.8)<br>41 (28-42)<br>3	79.1 (39.2)<br>62.5 (38-155)<br>16
Transferrin	23-45 µmol/L	n.a.	30.6 (1.1)<br>30.4 (29.6-31.7)<br>3
TfSat	23-46%	n.a.	29.7 (17.2)<br>27 (14-48.1)<br>3
Total bilirubin	<21 µmol/L	n.a.	6.8 (2.4)<br>7 (3-11)<br>14
GOT	<32 IU/L	22 (8.7)<br>17 (17-37)<br>5	17.6 (2.6)<br>17 (13-25)<br>18
GPT	<33 IU/L	n.a.	24.4 (32.5)<br>12 (9-101)<br>14
AP	<117 IU/L	n.a.	60.6 (7.5)<br>62.5 (47-67)<br>14
Gamma-GT	5-36 IU/L	n.a.	10.3 (1.2)<br>10 (9-12)<br>14

Table S1c: Laboratory parameters patient XLEPP3

XLEPP3 (m.)	Reference range	Before 2016<br>Mean (SD)<br>Median (range)<br>n	Since 2016<br>Mean (SD)<br>Median (range)<br>n
PPIX	<0.2 µmol/L	**22.5** (7.8)<br>**21** (15.6-30.9)<br>3	**12.5** (3.3)<br>12.6 (6.1-17.3)<br>8
ZnPP	<1.3 µmol/L	**10.9** (5)<br>**8.6** (7.4-16.7)<br>3	**10.3** (4)<br>**9.5** (6.2-18.2)<br>8
Relative ZnPP	%	32.8 (11.3)<br>32.3 (21.8-44.3)<br>3	44.6 (8.3)<br>44.7 (32.4-57)<br>8
Hb	13.5-17.2 g/dL	14.8 (0.5)<br>14.8 (14.4-15.1)<br>2	15 (0.7)<br>14.9 (13.9-16.1)<br>9
Platelets	150-370 10^3 /L	163 (38.2)<br>163 (136-190)<br>2	229.0 (33.8)<br>228 (185-275)<br>8
Eisen	6.6-26 µmol/L	n.a.	n.a.
Ferritin	30-400 µg/L	63<br>63<br>1	198.3 (88.2)<br>184.5 (91-377)<br>8
Transferrin	23-45 µmol/L	n.a.	n.a.
TfSat	16-45%	n.a.	n.a.
Total bilirubin	<21 µmol/L	n.a.	9 (2.1)<br>9 (5-13)<br>9
GOT	<50 IU/L	38.5 (0.7)<br>38.5 (38-39)<br>2	39.7 (34.1)<br>28 (19-127)<br>9
GPT	<50 IU/L	n.a.	**52.2** (51.8)<br>31.5 (17-186)<br>10
AP	40-129 IU/L	n.a.	60.5 (10.9)<br>58 (50-89)<br>10
Gamma-GT	<60 IU/L	n.a.	**128.2** (71.9)<br>113 (48-261)<br>10

Table S1d: Laboratory parameters patient XLEPP4

XLEPP4 (m.)	Reference range	Before 2016<br>Mean (SD)<br>Median (range)<br>n	Since 2016<br>Mean (SD)<br>Median (range)<br>n
PPIX	<0.2 µmol/L	**28.5** (12.6)<br>**28.3** (10.1-48.6)<br>6	**10.1** (2.8)<br>**9.9** (4.7-15.2)<br>16
ZnPP	<1.3 µmol/L	**14.7** (5.2)<br>**12.3** (10.3-22)<br>6	**9.4** (3.2)<br>**8.8** (4.5-15.5)<br>16
Relative ZnPP	%	36.3 (14.3)<br>25.6 (18.7-56.8)<br>6	48 (7.6)<br>47.1 (37.1-59.8)<br>16
Hb	13.5-17.2 g/dL	**12.9** (0.8)<br>**12.9** (11.7-14)<br>5	14.9 (0.7)<br>15 (13.5-15.9)<br>16
Platelets	150-370 10^3 /L	**107.4** (27.3)<br>**112** (79-144)<br>5	208.7 (38.5)<br>209 (113-268)<br>16
Eisen	6.6-26 µmol/L	n.a.	10.4 (1.9)<br>10.4 (9-11.7)<br>2
Ferritin	30-400 µg/L	**9.5** (0.7)<br>**9.5** (9-10)<br>2	102.5 (63.4)<br>86 (29-248)<br>15
Transferrin	23-45 µmol/L	n.a.	33 (1.2)<br>33 (23.1-33.8)<br>2
TfSat	16-45%	n.a.	**15.5** (3.5)<br>**15.5** (13-18)<br>2
Total bilirubin	<21 µmol/L	n.a.	6.1 (1.4)<br>6 (4-9)<br>11
GOT	<50 IU/L	22.6 (10.5)<br>19 (14-41)<br>5	17.9 (3.2)<br>18.5 (14-23)<br>16
GPT	<50 IU/L	n.a.	22.9 (6.3)<br>22 (10-34)<br>11
AP	40-129 IU/L	108<br>108<br>1	66.6 (6.9)<br>65 (58-81)<br>11
Gamma-GT	<60 IU/L	n.a.	18.9 (2.8)<br>18.5 (15-25)<br>14
